# Comparison of nutritional value of snakehead fish from Guangdong and Deqing varieties

**DOI:** 10.1371/journal.pone.0301203

**Published:** 2024-03-27

**Authors:** Zeguo Zeng, Miao Zheng, Mengxiao Zhao, Jing Guo, Shuifa Zhu, Xianguo Zou, Qingxiang Zeng

**Affiliations:** 1 Ganzhou Animal Husbandry and Fisheries Research Institute, Gannan Academy of Sciences, Ganzhou, 341000, People’s Republic of China; 2 Agricultural Technology Promotion Center of Deqing County, Deqing, 313200, People’s Republic of China; 3 College of Food Science and Technology, Zhejiang University of Technology, Hangzhou, 310000, People’s Republic of China; Tamil Nadu Dr J Jayalalithaa Fisheries University, INDIA

## Abstract

Environmental pollution and overfishing of wild fish resources have led to a significant decrease in snakehead fish, thus leading to the increased demand for breeding the snakehead fish. Guangdong and Deqing snakehead fish are two common consumed varieties. However, their nutritional value was unclear. Therefore, this study aimed to evaluate the nutritional value of snakehead fish from Guangdong and Deqing varieties feeding with different fodders by analyzing and comparing the proximate composition, fatty acids and amino acids. Results showed that the contents of carbohydrate, energy and fat contents in Guangdong variety were lower than that in Deqing variety feeding commercial fodder or offal. Besides, Guangdong variety contained the highest contents of polyunsaturated fatty acids (27.99 ± 1.99%) and EPA + DHA (2.70 ± 0.04%), as well as total essential amino acid content (2550.29), compared to Deqing variety feeding commercial fodder or offal. Overall, snakehead fish from Guangdong variety displayed the highest nutritional value, and thus was a reasonable choice for farmers and consumers. The findings of this study would help farmers to choose the suitable feeding variety and patterns of snakehead fish from the perspective of fish nutritional value, which is beneficial to the sustainable fish farming.

## 1. Introduction

Snakehead fish (*Channa argus*), commonly known as black fish, is a fierce carnivorous animal distributed in rivers, lakes and ponds. It is largely found in various water systems of India, Southeast Asia, Russia, Korea and Japan [1,2]. Especially, China has the large distribution, mainly found in the Yangtze River basin. Due to its rapid growth, strong adaptability, delicious taste, less muscular thorns, and abundant nutritional value, snakehead fish is welcomed by the majority of breeding farmers and consumers, and has become one of the important freshwater economic fish [3].

Amino acids and fatty acid compositions are two main nutrients and indicators reflecting the quality of snakehead fish [4]. Besides, snakehead fish was reported to contain rich long-chain n-3 polyunsaturated fatty acids (LC n-3 PUFA), especially eicosapentaenoic acid (EPA) and docosahexaenoic acid (DHA). LC n-3 PUFA was very useful for reducing obesity, including suppression of appetite, enhancement of fat oxidation and energy expenditure, and reduction of fat deposition [5]. EPA and DHA can help improve blood circulation and promote brain development, thus has the benefit to improve cardiovascular health and reduce cardiovascular disease (CVD) risk [6]. Snakehead fish is the good source of dietary EPA and DHA and deeply loved by the consumer [7,8].

The snakehead fish is an important food fish in China, and the Chinese tend to consume wild snakehead fish [9]. In recent years, due to overfishing, the wild resources of snakehead fish have decreased significantly, and a large scale of fish farming has emerged. In order to control the breeding cost and increase the income, the farmers often increase the breeding density and reduce the feed cost in the farming process, which inevitably brings about the decline of the quality of snakehead fish. Fish nutrition advanced dramatically with the application of new, balanced commercial diets that can promote optimal fish growth and health [10]. Some scholars have studied the influence of different feeds on the nutrition and behavior of snakehead fish [11,12]. Some scholars studied the influence of low salt environment on the growth and nutritional value of snakehead fish [13]. Besides, there was some research reporting the nutritional and commercial value of different varieties of snakehead fish [14].

Nevertheless, there were few studies on comparing the nutritional value of different varieties of farmed snakehead fish. Therefore, it is of significant importance to investigate the influence of different varieties on the nutritional value of snakehead fish. In this study, the nutritional components including protein, fat, fatty acid and amino acid were detected and compared. Results will help farmers choose suitable varieties to improve the quality of snakehead fish from the aspect of nutritional value.

## 2. Materials and methods

### 2.1 Sample information

Snakehead fish of two varieties: Guangdong variety and Deqing variety.

Deqing variety: feeding with commercial fodder (the puffed snakehead compound particle feed, Quanjiafu 8326, Jiangsu, China) or offal (the waste of animal viscera). Guangdong variety: feeding with commercial fodder (the puffed snakehead compound particle feed, Quanjiafu 8326, Jiangsu, China).

### 2.2 Determination of proximate composition

The chemical compositions (moisture, crude fat and crude protein) of all the samples were determined according to the AOAC (1990) procedures.

Moisture: samples were dried in oven at 103°C for 8 h; Crude fat was determined by gravimetric method after the Soxhlet extraction; crude protein (N * 6.25) (%) was detected by the Kjeldahl method after acid digestion.

The energy values were calculated using the mean values of protein and lipids in the snakehead fish according to the method reported by Usydus et al. [15]. The calculations were made with the following energy equivalents.

- protein-17 kJ/g
- lipids-37 kJ/g

### 2.3 Determination of fatty acids by GC-FID

Fatty acids were detected according to our previously published methods [16]. Approximately 25 ± 0.1 mg of lipid sample were inserted into a tube, and added with 500 μL of methyl tricosanoate (1 mg/mL) and 4 mL of a 0.5 mol/ L NaOH solution in methanol. Then the tube was closed and placed in an ultrasonic bath at room temperature (25°C) for 5 min. After that, 5 mL of esterifying reagent was added, and the tube was once again closed and placed in the bath for 6 min. Then, the tube was definitely removed from the bath and 4 mL of a saturated sodium chloride solution was added, and the entire system was closed and vigorously stirred for 30 s. Approximately 2 mL of n-hexane was added and then the tube was closed again and stirred for 30 s again. After 24 h of rest under -18 °C, the organic phase of tube was collected for chromatographic analysis. The chromatographic separation was performed using a molten quartz capillary column (100 m×0.25 mm×0.2 μm, CP-Sil 88, Chrompack; Agilent, USA). The carrier gas was H_2_, and the combustion gas was N_2_, H_2_ and air. The temperature procedure was 45°C for 4 min, increased up to 175°C at a rate of 13°C/min and maintained for 27 min, then further increased to 215°C at a rate of 4°C/min and held for 35 min, and the total running time was 86 min. The hydrogen flow rate was 30.0 mL/min, the air flow rate was 300 mL/min, and the nitrogen flow rate was 30.0 mL/min.

### 2.4 Determination of total amino acids (TAA) by HPLC

Snakehead fish was homogenized and dried at 105°C, ground into powder and passed through 40 mesh sieve. One gram of snakehead fish were added with 6 mol/L HCl, and then hydrolyzed at 110°C for 24 h. The hydrolysates were concentrated and dried by evaporation. Then the dried samples were dissolved in 0.02 M HCl (6 mL) and passed through a 0.22 μm filter membrane (Merck, Darmstadt, Germany) to remove impurities. The hydrolysates were separated and identified by a Model L-8900 Amino Acid Auto-Analyzer (L-8900, HITACHI, Japan) with analytical C18 column (4.6×150 mm, 5 μm, Agilent Technologies).

### 2.5 Calculation of amino acid scores (AAS), chemical scores (CS), and essential amino acid index (EAAI)

AAS and CS was calculated according to the equations of FAO/WHO (2013) [17], also reported by Oztekin et al. [18].

$$\text{AAS}={\text{AA}}_{\text{FBP}}/{\text{AA}}_{\text{FW}}$$

where, AA_FBP_ is the concentration level of amino acid per test protein (mg/g, FBP: fish body protein), and calculated as follows:

$${\text{AA}}_{\text{FBP}}=\left(\text{essential}\;\text{amino}\;\text{acid}/\text{crude}\;\text{protein}\right)\times 6.25\times 1000$$

and AA_FW_ shows the level of amino acid per protein with the reference to composition of FAO/WHO standard (mg/g) as given in Table 4.

$$\text{Chemical}\;\text{Score},\text{CS}=\left({\text{AA}}_{\text{FBP}}\right)/\left({\text{AA}}_{\text{EGG}}\right)$$

where, AA_EGG_ represents the concentration of amino acid per protein referred to the composition (mg/g) of whole egg protein (mg/g) as listed in Table 4.

The EAAI were calculated according to Oztekin et al. [18]:

$$\begin{array}{l} \text{EAAI}=\\ \sqrt[{\text{n}}]{\left[(100\times {\text{EAA}}_{1}/{\text{EAA}}_{1\text{EG}})\times (100\times {\text{EAA}}_{2}/{\text{EAA}}_{\text{2EG}})\times \ldots \times (100\times {\text{EAA}}_{\text{n}}/{\text{EAA}}_{\text{nEG}})\right]} \end{array}$$

where, “n” is the number of amino acids (considering pairs such as methionine + tyrosine)

EAA_1_, EAA_2_, … EAA_n_ are the levels of essential amino acids per test protein. EAA_1EG_, EAA_2EG_, …, EAA_nEG_ are the levels of essential amino acids per test protein of the egg reference concentration.

### 2.6 Statistical analysis

All tests were conducted in triplicate to minimize deviation, and data were presented as mean ± SD. The differences among different groups were compared using one-way ANOVA and Turkey HSD test with the help of the SPSS 17.0 software.

## 3. Results and discussion

### 3.1 Proximate composition

The basic nutrients of snakehead fish from Guangdong and Deqing varieties feeding with different fodders were shown in Table 1. Protein contents of snakehead fish in three groups were 17.40, 16.80 and 18.40 g/100 g respectively. It can be seen that snakehead fish of Deqing variety fed with offal had the highest protein content.

**Table 1:** Proximate composition of snakehead fish from different varieties and feeding with different fodders.

Proximate components	Snakehead fish from Guangdong variety (Commercial fodder)	Snakehead fish from Deqing variety (Commercial fodder)	Snakehead fish from Deqing variety (Feeding with offal)
Protein (g/100 g)	17.40^b^±0.21	16.80^b^±0.41	18.40^a^±0.31
Fat (g/100 g)	3.20^c^±0.10	6.20^b^±1.22	10.50^a^±0.71
Carbohydrate (g/100 g)	3.34^c^±0.12	4.27^b^±0.08	5.43^a^±0.31
Moisture (g/100 g)	68.03^b^±0.44	69.86^a^±0.43	70.32^a^±0.78
Energy (kJ/100 g)	414.00c±2.00	515.00^b^±0.34	701.00^a^±1.73

The fat content of snakehead fish from Guangdong variety feeding with commercial fodder was 3.20 g/100 g, significantly lower than those of Deqing variety feeding with two different fodders. This might be that the digestion and absorption of nutrients in different varieties are different [19,20]. Similarly, the energy in snakehead fish from Guangdong variety was also the lowest (414 kJ/100 g) compared to that from Deqing variety, no matter of the forage (515.00 and 701.00 kJ/100 g).

Besides, snakehead fish of Deqing variety feeding with offal had significantly higher fat content than Deqing variety feeding with commercial fodder (10.50 vs 6.20 g/100 g). This might be that the fat content in visceral fodder was higher than commercial fodder. Similarly, in three groups, the snakehead fish from Deqing variety feeding with offal had the highest carbohydrate content of 5.43 g/100 g.

### 3.2 Fatty acid compositions

Fatty acid content is an important indicator to evaluate the nutritional value of fish. Studies have shown that when the intake of unsaturated fatty acids is insufficient, it will cause cardiovascular and cerebrovascular diseases and tumors [21,22], and the loss of omega-3 fatty acids (especially EPA and DHA) will cause the lack of nutrients in the brain, thus affecting thinking and memory [23]. The fatty acids composition of three groups were shown in Table 2, the saturated fatty acids (SFA), monounsaturated fatty acids (MUFA) and PUFA were 30.59~33.13%, 41.49~51.55%, and 17.38~27.99%, respectively. Easy to see, MUFA accounted for the highest content among all fatty acids. The highest PUFA content (27.99±1.99%) was found in Guangdong variety feeding with commercial fodder. The MUFA in three groups showed opposite trend with PUFA. The MUFA of snakehead fish from Deqing variety was significantly higher than that from Guangdong variety (41.49±1.21%), and the highest MUFA content was found in Deqing variety feeding with offal (51.55 ± 0.29%).

**Table 2:** Fatty acid compositions (%) of snakehead fish from different variety and feeding with different fodders.

Fatty acids	Snakehead fish from Guangdong variety (Commercial fodder)	Snakehead fish from Deqing variety (Commercial fodder)	Snakehead fish from Deqing variety (Feeding with offal)
C10:0	0.05^a^±0.01	0.02^b^±0.01	0.02^b^±0.01
C12:0	0.05^a^±0.00	0.03^b^±0.00	0.03^b^±0.01
C14:0	0.82^a^±0.23	0.43^b^±0.17	0.35^c^±0.12
C14:1	-	-	0.02±0.00
C15:0	0.12^a^±0.08	0.06^b^±0.05	0.05^b^±0.01
C16:0 (PA)	22.80^c^±0.11	24.00^a^±0.02	23.40^b^±0.03
C16:1	3.27^a^±0.75	2.45^b^±0.06	2.32^b^±0.04
C17:0	0.23^a^±0.04	0.16^b^±0.01	0.15^b^±0.03
C17:1	0.11±0.06	0.12±0.06	0.11±0.04
C18:0	5.69^b^±0.07	6.95^a^±0.13	5.71^b^±0.09
C18:1(OA)	36.10^c^±0.31	45.80^b^±0.04	48.00^a^±0.14
C18:2(LA)	21.40^a^±1.33	9.26^b^±0.46	10.10^b^±0.52
C18:3 n-6	0.28^a^±0.15	0.22^b^±0.09	0.28^a^±0.10
C18:3 n-3	1.18^a^±0.43	0.24^b^±0.09	0.30^b^±0.09
C20:0	0.24^a^±0.07	0.11^b^±0.06	0.08^b^±0.01
C20:1	1.81±0.08	1.09±0.06	1.05±0.09
C20:2	0.51^a^±0.09	0.30^b^±0.16	0.31^b^±0.13
C20:3 n-6	0.30^c^±0.05	0.63^a^±0.03	0.50^b^±0.07
C20:4	1.52^b^±0.07	5.93^a^±1.11	5.55^a^±1.35
C20:5(EPA)	0.30^a^±0.02	0.10^b^±0.01	0.03^c^±0.00
C21:0	0.16^b^±0.06	0.46^a^±0.02	0.45^a^±0.14
C22:0	0.09^a^±0.01	0.06^b^±0.00	0.03^c^±0.00
C22:1	0.15^a^±0.01	0.05^b^±0.00	0.04^b^±0.00
C22:2	0.08^a^±0.00	0.03^b^±0.01	0.02^b^±0.00
C24:0	0.34^b^±0.05	0.83^a^±0.11	0.74^a^±0.09
C24:1	0.04±0.01	0.03±0.00	0.02±0.00
C22:6(DHA)	2.40^a^±0.02	0.67^b^±0.04	0.34^c^±0.01
SFA	30.59^b^±0.58	33.13^a^±0.32	31.00^b^±0.24
MUFA	41.49^c^±1.21	49.63^b^±0.07	51.55^a^±0.29
PUFA	27.99^a^±1.99	17.38^b^±1.08	17.43^b^±1.58
TUFA	69.48^a^±2.88	67.00^b^±1.10	68.98^a^±1.47
EPA+DHA	2.70^a^±0.04	0.77^b^±0.04	0.37^c^±0.01

Note: *for saturated fatty acids, # for monounsaturated fatty acid, & for polyunsaturated fatty acid; ‐ represents not detected; TUFA, total unsaturated fat acids; the different letters in the same line indicated significant differences (*p* < 0.05).

The content of C18:2 (LA) in Guangdong variety was 21.40±1.33%, significantly higher than that in Deqing variety (9.26±0.46% and 10.10±0.52%). The content of LA in Deqing variety feeding with different fodders showed no significantly difference. The results showed that the variety (gene difference) rather than the type of fodders play an important influence in the content of LA. The amount of C18 fatty acids (such as OA, LA and ALA) was reported to have high nutritional value because they protect against cardiovascular disease (CVD) and contribute to the enrichment of aromatic components [24–26].

Numerous studies have shown that DHA plays an important role in normal retina and brain development [23]. Although LA can be converted into EPA in the human body, the speed of this reaction in the human body is very slow and the amount of conversion is very small, far from meeting the human body’s needs [27]. Therefore, it must be directly supplemented from food to meet body’s need. Fish is a good food source for DHA and EPA. The American Heart Association (AHA) suggests that people who are diagnosed with coronary heart disease (CHD) should intake approximately 1 g of DHA and EPA every day [28]. People without CVD should intake approximately 500 mg of these acids each day for prophylactic purposes [29,30]. Higher doses of DHA and EPA were reported to decrease high triglycerides levels in the blood [31]. The AHA suggests that a daily intake of approximately 2 ~ 4 g of these acids can lower triglycerides. The contents of EPA and DHA of snakehead fish from Guangdong variety (2.70±0.04%) were significantly higher than that from Deqing variety (0.37±0.01% and 0.77±0.04%), suggesting that snakehead fish of Guangdong variety was the good source of EPA and DHA.

### 3.3 The amino acid composition

As shown in Table 3, a total of 16 amino acids were detected in snakehead fish, including 7 essential amino acids (EAA), 9 non-essential amino acids (NAA). The type and content of amino acids can reflect the quality of food protein, and the content of total EAA (TEAA) is the most important index to evaluate the nutritional value [32]. In the present study, the TAA and TEAA content of snakehead fish from Guangdong variety and from Deqing variety feeding with offal were not significantly different, and both were significantly higher than that from Deqing variety feeding with commercial fodder. The result suggested that the varieties and the types of fodders both demonstrated great difference in amino acids content of snakehead fish.

**Table 3:** Amino acid compositions of snakehead fish from different varieties and feeding with different fodders (g/100 g).

	Snakehead fish from Guangdong variety (Commercial fodder)	Snakehead fish from Deqing variety (Commercial fodder)	Snakehead fish from Deqing variety (Feeding with offal)
EAA (g/100g)			
Lys	1.84±0.55	1.62±0.36	1.85±0.42
Thr	0.82±0.31	0.70±0.41	0.78±0.24
Val	0.78^b^±0.09	0.70^c^±0.41	0.86^a^±1.02
Met	0.34^b^±0.31	0.32^b^±0.11	0.44^a^±0.23
Ile	0.82^b^±0.09	0.72^b^±0.14	0.86^a^±0.08
Leu	1.34^a^±0.13	1.17^b^±0.46	1.38^a^±0.31
Phe	0.74^a^±0.09	0.64^b^±0.19	0.74^a^±0.16
TEAA (g/100g)	6.68^a^±1.44	5.87^b^±1.44	6.91^a^±0.51
NEAA (g/100g)			
Glu	3.38^a^±0.51	2.68^b^±0.31	3.00^a^±0.43
Asp	1.80±0.09	1.52±0.21	1.72±0.37
Ala	1.31^b^±0.41	1.23^b^±0.52	1.36^a^±0.32
Gly	0.82±0.10	0.80±0.12	0.83±0.09
Ser	0.76^a^±0.42	0.60b±0.24	0.66b±0.22
Pro	0.41±0.09	0.46±0.08	0.45±0.06
Tyr	0.42^a^±0.01	0.35^b^±0.01	0.44^a^±0.01
His	0.46±0.09	0.44±0.09	0.48±0.07
Arg	0.90^a^±0.14	0.79^b^±0.27	0.90^a^±0.32
TNEAA (g/100g)	10.26^a^±0.70	8.87^b^±0.91	9.83^a^±0.38
TAA (g/100g)	16.94^a^±0.93	14.74^b^±0.61	16.73^a^±0.61
TFAA (g/100g)	7.31^a^±0.53	6.23^c^±0.90	6.90^b^±0.22
Ratios (%)			
TEAA/TAA	39	40	41
TEAA/TNEAA	65	66	70
TFAA/TAA	43	42	41

Note: EAA, essential amino acids; TEAA, total essential amino acids; NEAA, non essential amino acids; TNEAA, total non essential amino acids; TAA, total amino acids; TFAA, total flavor amino acids (Glu, Asp, Ala and Gly); the different letters in the same line indicated significant differences (*p* < 0.05).

Flavor amino acids (TFAA) include Glu, Asp, Ala and Gly, which determines the flavor taste of the food protein. Glu and Asp are umami amino acids, while Gly and Ala are sweet amino acids. As listed in Table 3, the TFAA content from Guangdong variety (commercial fodder) was the highest. It is worth noting that the content of TAA, TEAA and TNEAA from Guangdong variety feeding with commercial fodder was significantly higher than those from Deqing variety feeding with commercial fodder, but there is no significant difference between Guangdong variety feeding with commercial fodder and Deqing variety feeding with offal. These results suggested that the fodders and the cultivating varieties both can influence the TAA, TEAA and TNEAA in snakehead fish.

### 3.4 Protein and nutritional quality evaluation

According to the FAO/WHO amino acid pattern, the TEAA/TAA value is about 40%, and the TEAA/TNEAA value should exceed 60%. In this study, the TEAA/TAA value of three groups were 39%, 40% and 41% respectively, and the TEAA/TNEAA value were 65%, 66% and 70%. These results suggested that the snakehead fish of three groups met the FAO/WHO amino acid pattern standard and belong to the high quality protein source food.

As shown in Table 4, the total essential amino acid content in three groups (2550.29, 2314.00 and 2496.62) were higher than that at FAO/WHO amino acid pattern (2190), and Guangdong variety showed the highest. According to AAS and CS, content of Lys in all three groups (660.92, 602.68 and 628.40) far exceeded the requirement of FAO/WHO amino acid pattern (340) and whole egg amino acid pattern (441), especially Guangdong variety. The first limiting amino acid was Met, both in Guangdong and Deqing varieties. Notably, EAAI value in Guangdong variety (79.08) was higher than Deqing variety feeding with commercial fodder (72.24), and was similar with Deqing variety feeding with offal (79.23). The results were in agreement with that of amino acid compositions. Higher EAAI indicates more reasonable amino acid composition, better protein quality and higher utilization [33,34]. The mixtures of branched-chain and aromatic amino acids have the liver protection effect [35,36], and the F value (molar ratios of branched-chain amino acids to aromatic amino acids) of normal people is 3.0 ~ 3.5, when the liver is damaged, it is reduced to 1.0 ~ 1.5. In this study, the F values of three groups were 2.52, 2.62 and 2.63.

**Table 4:** Amino acid value of snakehead fish from different varieties and feeding with different fodders.

	AA_FBP_ (mg/g)					SFGD (CF)		SFDQ (CF)		SFDQ (FWO)	
	SFGD (CF)	SFDQ (CF)	SFDQ (FWO)	F/W standard(mg/g)	WEP standard (mg/g)	AAS	CS	AAS	CS	AAS	CS
Thr	294.54	260.42	264.95	250	292	1.18	1.01	1.04	0.89	1.06	0.91
Val	280.17	260.42	292.12	310	410	0.9	0.68	0.84	0.64	0.94	0.71
Leu	481.32	435.27	468.75	440	534	1.09	0.9	0.99	0.82	1.07	0.88
Ile	294.54	267.86	292.12	250	331	1.18	0.89	1.07	0.81	1.17	0.88
Lys	660.92	602.68	628.4	340	441	1.94	1.5	1.77	1.31	1.85	1.42
Met	122.13	119.05	149.46	220	386	0.56	0.32	0.54	0.31	0.68	0.39
Phe+Tyr	416.67	368.30	400.82	380	565	1.1	0.74	0.97	0.65	1.05	0.71
Total	2550.29	2314.00	2496.62								
EAAI	79.08	72.24	79.23								
F	2.53	2.62	2.63								

Note: SFGD (CF), snakehead fish from Guangdong variety (Commercial fodder); SFDQ (CF), snakehead fish from Deqing variety (Commercial fodder); SFDQ (FWO), snakehead fish from Deqing variety (Feeding with offal); F/W standard, FAO/WHO standard; WEP standard, whole egg protein standard; AA_FBP_, fish body protein amino acids; EAAI, essential amino acid index; AAS, amino acid score; CS, chemical score.

To sum up, in the same cultivating condition, Guangdong variety displayed the best nutritional value. Offal fodder could improve the nutritional quality of snakehead fish from Deqing variety.

## 4. Conclusions

In general, this study provides a detailed analysis and comparison of the nutritional composition of snakehead fish among Guangdong variety and Deqing variety feeding with different fodders, including proximate components, fatty acids and amino acids. The Guangdong variety had the lowest carbohydrate, energy and fat contents. Besides, the total amount of PUFA and EPA + DHA in the Guangdong variety was the highest, as well as the essential and flavor (Glu, Asp, Ala and Gly) amino acids, implying that Guangdong variety could be beneficial for decreasing blood lipid and preventing atherosclerosis. Therefore, Guangdong variety showed better nutritional quality than Deqing variety, and suitable fodder can improve the nutritional value of snakehead fish. From the perspective of nutritional value, this study would provide a theoretical basis for farmer to choose suitable variety and feeding patterns.

## Supporting information

S1 Dataset (XLSX)
